# The role of different components of the immune system against *Plasmodium falciparum* malaria: Possible contribution towards malaria vaccine development

**DOI:** 10.1016/j.molbiopara.2021.111425

**Published:** 2021-11

**Authors:** Wilson L. Mandala, Visopo Harawa, Fraction Dzinjalamala, Dumizulu Tembo

**Affiliations:** a Academy of Medical Sciences, Malawi University of Science and Technology, Thyolo, Malawi; b Malawi Liverpool Wellcome Trust, Blantyre, Malawi

**Keywords:** Malaria, Immune cells, Vaccine candidates

## Abstract

*Plasmodium falciparum* malaria still remains a major global public health challenge with over 220 million new cases and well over 400,000 deaths annually. Most of the deaths occur in sub-Saharan Africa which bears 90 % of the malaria cases. Such high *P. falciparum* malaria-related morbidity and mortality rates pose a huge burden on the health and economic wellbeing of the countries affected. Lately, substantial gains have been made in reducing malaria morbidity and mortality through intense malaria control initiatives such as use of effective antimalarials, intensive distribution and use of insecticide-treated nets (ITNs), and implementation of massive indoor residual spraying (IRS) campaigns. However, these gains are being threatened by widespread resistance of the parasite to antimalarials, and the vector to insecticides. Over the years the use of vaccines has proven to be the most reliable, cost-effective and efficient method for controlling the burden and spread of many infectious diseases, especially in resource poor settings with limited public health infrastructure. Nonetheless, this had not been the case with malaria until the most promising malaria vaccine candidate, RTS,S/AS01, was approved for pilot implementation programme in three African countries in 2015. This was regarded as the most important breakthrough in the fight against malaria. However, RTS,S/AS01 has been found to have some limitations, the main ones being low efficacy in certain age groups, poor immunogenicity and need for almost three boosters to attain a reasonable efficacy. Thus, the search for a more robust and effective malaria vaccine still continues and a better understanding of naturally acquired immune responses to the various stages, including the transmissible stages of the parasite, could be crucial in rational vaccine design. This review therefore compiles what is currently known about the basic biology of *P. falciparum* and the natural malaria immune response against malaria and progress made towards vaccine development.

## Highlights

• Although substantial gains have been made in reducing malaria burden through various initiatives, it is still a major public health challenge.
• Unlike other infectious diseases, there had not been any malaria vaccine until RTS,S/AS01 was approved for pilot trial in three countries in 2015.
• However, RTS,S/AS01 still has low efficacy in some age groups, poor immunogenicity and requires three boosters to attain a reasonable efficacy.
• The search for a more effective malaria vaccine therefore still continues, hence the need for a better understanding of the host immunity.
• This review therefore compiles what is known about the basic biology of *P. falciparum*, the host immune response and progress on malaria vaccines.

## 1 Introduction

Malaria is a mosquito borne infectious disease of humans and animals, which is caused by a protozoan parasite of genus *Plasmodium* [1]. Five species of *Plasmodium* are now known to cause human malaria namely; *Plasmodium vivax, P. malariae, P. ovale* and *P. falciparum* and *P. Knowlesi,* [1,2]. In 2019, the World Health Organisation (WHO) estimated the malaria burden at about 229 million cases and 409,000 deaths worldwide, with *P. falciparum* causing the vast majority of the cases of which 94 % occurred in Africa [1]. *Plasmodium* parasites are transmitted from one person to another by female *Anopheles* mosquitoes. Of the 400 species of *Anopheles* mosquito; only 30 transmit malaria with *An. gambiae s.s*, *An. arabiensis* and *An. Funestus* being the three main vectors commonly found in Sub-Saharan Africa (SSA) [1,3].

## 2 *P. falciparum* life cycle

**Fig. 1:**
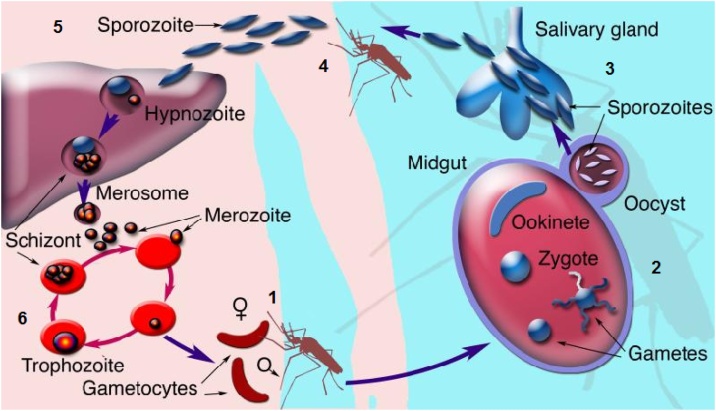
Life cycle of *P. falciparum* showing the various stages (arbitrarily labelled 1 to 6). Various vaccine candidates have been developed and are in the process of being tried. For Stage 1 (Pf25, Pf230, Pfg27, Pfs45/48, Pfs16, Pfs28. For Stage 4, CSP-1, TRAP, STARP, SALSA, SSP-2, for Stage 5, Attenuated sporozoites RTS,S and/or AS02 with prime boost (ME-TRAP), SPF66, MuSTDO. For Stage 6 (Ring Stage: Combination B (MSP-1, MSP-2, RESA), MSP-1 and/or AS02, MSP-3 and or GLURP, AMA-1.) (Trophzoite Stage: SERA, AMA-1, RAP-1, MSP-1, MSP-2, MSP-3, MSP-5, EBA-175, RAP-2, GLURP, RESA, EMP-1, Pd35, Pf55, PfRH5). List of vaccine candidates adapted from Tongren et al., [177]. **Abbreviations**: AMA, apical membrane antigen; CSP, circumsporozoite surface protein; EBA, erythrocyte-binding antigen; EMP, erythrocyte membrane protein; GLURP, glutamate-rich protein; ME–TRAP, multiple epitope–thrombospondin-related adhesive protein; MSP, merozoite surface protein; Pf, P. falciparum protein; RAP, rhoptry-associated protein; RESA, ring-infected erythrocyte surface antigen; SALSA, sporozoite- and liver-stage antigen; SERA, serine-repeat antigen; SPf66, synthetic P. falciparum 66; SSP, sporozoite surface protein; STARP, sporozoite threonine- and asparagine-rich protein; TRAP, thrombospondin-related adhesive protein. Figure adapted from Winzeler, [19].

One of the main factors that have made *P. falciparum* such a formidable parasite for humans to deal with, and also cause challenges in malaria vaccine development, is the complicated parasite’s life cycle (Fig. 1) shared by all *Plasmodium* species, which involves both a vertebrate host and an insect vector [4]. The life cycle begins when a female anopheles mosquito takes a blood meal from an infected human being thereby ingesting infected red blood cells (iRBCs) containing gametocytes. The male and female gametocytes combine in the mosquito gut to form a zygote [[5], [6], [7]] which develops into an ookinete that migrates through the mosquito midgut epithelium to become an oocyst between 24−36 hours, then further develops into sporozoites through asexual sporogenic replication [8].

When the infective mosquito has its next blood meal, sporozoites migrate to the vector’s salivary glands and are inoculated into the skin of a bitten person *via* the mosquito’s saliva and the sporozoites can remain under the skin for as long as six hours before entering the blood stream [6]. Although only less than 100 sporozoites are inoculated per infective mosquito bite [5], only a third of these manage to reach the liver [9]. The number of inoculated sporozoites does not influence the disease outcome but is known to affect the time before malaria-related symptoms appear [8], making this stage crucial and as such a target for a number of vaccine candidates, including the RTS,S [10]. Within two to thirty minutes sporozoites reach and infect liver cells where they multiply to form merozoites [6]. Considering that one sporozoite can potentially reproduce asexually to form up to 40,000 merozoites, it is in the interest of the host’s immune system to deal with this stage of the life cycle urgently to minimize a serious challenge on the immune system imposed by the merozoites. The liver stage lasts between two and ten days after which the infected liver cells bursts, releasing a huge number of merozoites which infect red blood cells (RBCs) [5]. This process of asexual replication in hepatocytes, known as schizogony, is part of the pre-erythrocytic stage of the parasite’s life cycle [6,11,12].

The erythrocytic stage commences once the merozoites leave the liver cells and enter the bloodstream, infect RBCs and begin to develop into trophozoites and finally schizonts, which rupture and release more merozoites that attack uninfected RBCs. Each schizont can contain up to thirty-six merozoites [11]. The erythrocytic cycle lasts for 48 h and it is during this stage that an individual would start to experience malaria-related symptoms such as periodic fever upon each bursting phase of the RBCs to release the merozoites. The fever decreases during the parasite replication phase inside the RBCs, and the patient appears to be improving, [13].

During the third stage, also known as the sexual stage, some merozoite-infected RBCs cease reproducing asexually after several cycles of intraerythrocytic propagation and instead differentiate into sexual forms of the parasites as either male or female gametocytes (gametocytogenesis) [7,14,15]. Gametocytes sequester and mature in the bone marrow and enter peripheral circulation after some days where they are taken up by an Anopheles mosquito during a blood meal [[6], [7], [8]] recommencing the cycle. Sequestration of gametocytes in bone marrow is an immune evasion mechanism to avoid splenic clearance during development [11,12] and provides another potential area of disturbing the parasite’s life cycle.

## 3 Host immune response to malaria

Natural protective immunity against malaria takes years to develop, even in high transmission areas despite repeated exposure to the parasite. Apart from natural acquired immunity being parasite specific (with specific immune response activated and developed against specific strains of *P. falciparum* antigens [16]) it is also stage-specific and antigen specific such that the naturally acquired immunity developed against erythrocytic stage of the infection is not effective against the sporozoite stage or the intrahepatic stage [17,18]. Furthermore, antimalarial immunity is also influenced by age (with children aged five or younger residing in endemic areas being more vulnerable), genetics, pregnancy, nutritional status and co-infections [17]. However, regardless of how robust the host immune response that develops over time, it is easily breached during subsequent infections due to the complex life cycle of the *Plasmodium* parasite involving multiple organs (Fig. 1) [19].

Since the parasite is confined within the RBCs during the erythrocytic stages, it would therefore end up being transported to different body organs as the RBCs migrate. This being the case, immune mechanisms directed against the iRBCs can therefore easily affect many host organs. A detailed understanding of host immune mechanisms against *P. falciparum* parasites and how the parasite’s own mechanisms at different stages of its life cycle evade host immunity and how these immune mechanisms affect host tissues and how the mechanisms are regulated, is fundamental, not only in vaccine development, but also in treating people affected by the disease [20].

Although *Plasmodium* has been in existence for well over 5000 years, its complex life cycle is matched with an equally complex response from various effector functions of the host immune system which involves different cell types, malaria-specific antibodies and various cytokines. These various components of the immune response operate in a well-coordinated manner at different stages of the parasite’s life cycle [21].

### 3.1 Immune mechanisms against the pre-hepatic *P. falciparum* infection stage

The importance of antibodies in controlling sporozoites was first demonstrated in rodent malaria where immunisation with attenuated *Plasmodium berghei* sporozoites induced neutralising antibodies against sporozoites [22]. The replication of such experiments in human has confirmed the sporozoites-induced antibody production [23] even in naturally occurring infection in malaria endemic countries [[24], [25], [26]]. Although antigen-specific monoclonal antibodies have been shown to be effective in preventing *P. falciparum* sporozoites invading hepatocytes *in vitro* thereby stopping their development within the hepatocytes [27], antibodies on their own cannot be considered to be the solution to the problem. This is the case because sporozoites usually invade hepatocytes within 2−30 min after inoculation and the antisporozoite antibodies must be present in circulation at high titres and exert their activity within minutes of infection to prevent hepatocyte infection [21]. Because of this mismatch in timing antibody-mediated protective immunity against the sporozoite stage is not always effective even if the antibodies are eventually produced in large quantities. Sporozoites that have not been blocked by antibodies will infect host liver cells within minutes of inoculation during the pre-erythrocytic stage [17].

Three important antigens at this stage of the parasite are recognised by antibodies in sera from individuals from high malaria endemic area. Thrombospondin-related adhesive protein (TRAP) and circumsporozoite protein (CSP), both of which have been considered as vaccine candidates, and Liver Stage Antigen 1 (LSA1). Antibody responses to CSP, TRAP and LSA1 inhibit sporozoites invasion of hepatocytes and protect against infection, and reduce the risk of clinical malaria [28,29]. The RTS,S, the most advanced malaria vaccine consists of CSP antigen which causes the production of antibodies capable of preventing sporozoites invasion of hepatocytes and also elicits a cellular response enabling the destruction of infected hepatocytes [10]. Overall, studies about antibodies against sporozoite antigens reveal an age and dose-dependent magnitude that correlates with partial protection in some areas, but not sterile immunity.

### 3.2 Immune mechanisms against the Pre-Erythrocytic stage

*P. falciparum* developing within hepatocytes are the main target of protective immunity directed against the pre-erythrocytic stage. Both CD8+ and CD4 + T cells recognise parasite-derived peptides presented by major histocompatibility complex (MHC) class I or class II molecules respectively, on the surface of infected hepatocytes. However protection against the pre-erythrocytic stage appears to be primarily mediated by CD8 + T cells as demonstrated in mice models in which *in vivo* depletion of CD8 + T cells abrogates protection and adoptive transfer of CD8 + T cells to naïve mice confers protection [30].

Protection against pre-erythrocytic stage malaria is in part mediated directly by CD8+ cytotoxic T lymphocytes (CTLs) with cytokines and other factors such as nitric oxide. *In vitro* treatment of *P. falciparum* infected hepatocytes with interferon gamma (IFN-γ) can eliminate the parasite from *in vitro* culture. In addition, IFN-γ has been linked with inducing production of nitric oxide *in vitro* and *in vivo* after infection with *P. falciparum* sporozoites [31]. Thus, natural exposure to parasite antigens may not be sufficient to induce robust T-cell responses against pre-erythrocytic stages partly due to the low antigen inoculum estimated at 10–100 sporozoites per mosquito bite, which may not be sufficient to induce a strong immune response as evidences. In support of this, it requires an intravenous injection of several hundred thousand sporozoites to produce a high degree of sterile protection [32].

### 3.3 Immune mechanisms against the Erythrocytic stage

Parasites develop within RBCs, which do not express MHC molecules. However, iRBCs express parasite-encoded variant surface antigens (VSAs) with *Plasmodium falciparum* erythrocyte membrane protein (PfEMP1) being the best characterised antigen [[18], [19], [20]]. Approximately 60 variant gene copies for PfEMP1 occur in each parasite capable of producing numerous PfEMP1 sequence diversity that mediate binding to different host tissue *via* different host receptors through different domains [[33], [34], [35]]. Clearance of a parasite clone appears to follow the development of a VSA-specific antibody response to that particular clone such that other parasites that switched expression to a different VSA survive and multiply. Furthermore, the new clone may possess different tissue adhesion phenotype altering pathology [36]. Both intra- and inter- clonal properties of VSA can potentially affect the development of one erythrocytic stage vaccine.

Products of ruptured iRBCs comprising a complex mixture of glycoproteins and glycolipids with endotoxin-like properties, with glycosylphosphatidyinositols (GPI) as the main component, can directly induce production of tumour necrosis factor alpha (TNF-α) and IFN-γ *via* an innate pathway comprising natural killer (NK) cells, macrophages and gamma/delta (γδ) T cells. At the same time antigen-specific alpha/beta (αβ) T cells are primed [37,38]. The low levels of IFN-γ and TNF-α produced at this stage are associated with inhibition of parasite replication thereby reducing parasitaemia levels and this presents another layer of malaria disease prevention.

Although CD8+ and CD4 + T cells have a protective role against the liver stage of malaria, results of earlier depletion and adoptive transfer experiments in mice models seemed to suggest that these cells had a limited role or even no role in protection against blood stages [39]. Instead, CD8+ and CD4 + T cells were previously implicated as the main mediators of experimental cerebral malaria (ECM) caused by various strains of *P. berghei* in mice. Further murine studies had confirmed that both CD4+ and CD8 + T cells are required for the development of ECM with CD4 + T cells involved in priming and CD8 + T cells as the effectors of ECM [31] and that the sequestration of activated CD8 + T cells in the cerebral microvasculature of mice contributed to the observed neurological symptoms and the resulting high mortality in the affected mice [40]. Researchers have recently shown the presence of CD8 + T cells in the brain of post-mortem fatal pediatric human cerebral malaria cases from Malawi with higher numbers of CD8 + T cells detected in those patients co-infected with HIV suggesting that HIV con-infection can influence the clinical outcome of cerebral malaria [41].

Work done in mice models has also shown that in addition to CD4+ and antibodies, CD8 + T cells, also play a major role in controlling *P. yoelli* infection [42]. These CD8 + T cells were characterised as actively proliferating cells which showed IL-7R and PD-1 expression. In contrast, in humans, it is the *P. vivax*, and not the *P. falciparum,* which is associated with the expansion of CD8 + T cell which is known to have cytotoxic feature [43].

In addition to parasitized RBCs, some have found monocytes and macrophages sequestered in the cerebral blood vessels in mice studies [44,45]. The observation of decreased proportion of non-classical monocytes in periphery was associated with death of CM cases suggesting that this subset of monocytes has a role in resolving malaria [46]. However, it is worth noting that even though the recruitment of activated monocytes and macrophages to the site of infection is essential for clearance of malaria infection, these two cell types have also been associated with adverse clinical outcome especially in CM cases due to the tendency of activated phenotypes to migrate to and sequester in cerebral microvasculature [47].

### 3.4 Immune mechanisms against the Gametocyte Stage of *P. falciparum* malaria

As mentioned before, the early stages of asexual parasites-infected RBCs (aiRBC) are found in circulation and as the parasite matures within the RBC it exports a large number of proteins to the host cell modifying its physical and immunogenic properties [48]. A sub-population of asexual blood-stage parasites differentiates into the sexual mosquito-transmissible form within RBCs, and within these gametocyte-infected RBCs (giRBC), the parasite develops through five distinct morphological stages (stage I-V). Like mature aiRBCs, immature giRBCs (stage I-III) express parasite proteins on the host cell [49] and sequester at several sites in the host, predominantly the bone marrow and spleen [50]. Upon maturation (stage-V) the gametocytes return to circulation where they can be taken up by female anopheline mosquitoes during a blood meal where they can develop further. However, while they are still in peripheral circulation the human host mounts an immune response against the gametocytes. Firstly naturally acquired antibodies against a number of gametocyte antigens such as Pfs230 and Pfs45/48 and the zygote/ookinete proteins Pfs25 and Pfs28 have been reported before [[51], [52], [53], [54]]. Secondly, CD8 + T cells [55] and γδ T [56] cells have been reported to play a role in the body’s immune response against gametocytes with CD4 + T cells being observed as the main producers of the cytokines TNF-α and IFN-γ that work directly against gametocytes [54].

## 4 The role of different cells in response to *P. falciparum* infection

Both cell-mediated and humoral-mediated immunity play important roles in defence mechanisms against malaria primarily depending on early cell-mediated innate responses and activation of antigen-specific T cell. Neutrophils, monocytes and NK cells all play some role in innate immunity experience earlier on during infections [11,13]. Natural killer T (NKT) cells have also been implicated in innate malarial immunity as potent inhibitors of liver stage parasite replication in mouse malaria systems *in vitro* [57].

### 4.1 Macrophages, monocytes and dendritic cells

Macrophages play an important role in eliminating iRBCs from circulation by phagocytosis [58]. Haemozoin, a polymerised form of haeme and other soluble endotoxins released from rapturing schizonts, have been shown to stimulate macrophage directly to produce TNF-α [59]. Haemozoin is rapidly ingested by monocytes and inhibits their maturation and differentiation to dendritic cells [60]. Ingestion of small numbers of iRBCs containing haemozoin inhibits further phagocytosis of iRBCs or other substrates. Furthermore, haemozoin inhibits oxidative burst and Protein Kinase C (PKC) activity [61,62], and the intracellular killing of pathogens by generation of superoxide radicals is greatly affected [63].

Dendritic cells (DCs) are antigen-presenting cells (APCs) and are not only crucial for the induction of primary immune responses, but may also be essential for the induction of immunological tolerance and regulation of T cell-mediated immune response [64]. DCs have been found to play an important role in initiating immune responses against malaria [65]. Recent work in *P. yoelli* has shown that mature CD11c + DCs are responsible for the initial priming of CD8 + T cells in the skin-draining lymph nodes [66] and DCs are also known to play a major role in internalising *Plasmodium* antigen, processing it and presenting it to CD4 + T cells at the early stages on CD4 + T cells activation.

### 4.2 CD8+ T cells in *P. falciparum* malaria

Previous studies conducted in mice-models, non-human primates and humans have shown that CD8 + T cells are the primary effector cells against pre-erythrocytic stages of various species of *Plasmodium* malaria [[67], [68], [69], [70], [71]]. Although exposure of humans and animals to sporozoites activates CD8 + T cells specific for antigens expressed in pre-erythrocytic stages [16], CD8+ cells have been shown to be crucial cytotoxic immune cells for eliminating parasites that successfully invade and replicate within hepatocytes [72]. However, studying CD8 + T cells and other cells as anti-parasitic immune cells against *P. falciparum* malaria in humans has ethical limitations which makes the use of mice models a better option.

Studies in *P. berghei* and *P. yoelii* mice models therefore have showed that cloned CD8 + T cells collected from immune mice and transferred into mice subsequently challenged with viable sporozoites are able to inhibit the development of liver stage parasites, thereby preventing subsequent RBCs infection [73]. This protective activity is proven to be stage-specific because transfer of these CD8 + T cells did not protect mice challenged with iRBCs. The number and quality of T cells required to achieve this protection was found to be within the physiological range of a normal immune response [30].

Naïve CD8 + T cells have been shown to be incapable of exerting anti-parasitic activity only attaining some ability to do so after being primed by APCs [74]. Work in *P.yoelii* has shown that initially CD8 + T cells are primed by mature CD11c + Dendritic cells in the skin-draining lymph nodes as already mentioned [66]. Unlike memory cells, naïve CD8 + T cells cannot eliminate parasitised cells immediately after antigen recognition but require a priming period for as long as twenty-four hours. Once primed, the cells show clear signs of differentiation and produce the mediators IFN-γ and perforin, and have the capacity to eliminate malaria parasites during the liver stage [30]. Proper development of a CD8 + T cell-response is greatly dependent on CD4 + T cells, since elimination of CD4 + T cells in mouse models reduced CD8 + T cell-response by more than 90 %. IL-4 secreted by CD4 + T cells is thought to be required for the full development of the CD8 + T cells response, although other cytokines such as IL-2, IL-15 and IL-17 may also play important roles [73,75].

Recent studies have shown that the primed CD8 + T cells differentiate to either short-lived effector cells (SLECs) or memory precursor effector cells (MPECs) subject to the cytokine environment and transcriptional factor profile [76,77]. SLECS and MPECs then undergo clonal expansion in the presence of CD4+Tcells- produced IL-2 or IL-4 as evidenced by a remarkable increase of these cells after sporozoites inoculation [66,42]. Eventually SLECs migrate to the liver to exert their effector properties in that organ whereas the MPECs undergo further differentiation to form proper and species-specific memory CD8 + T cells [78,79].

Malaria-specific memory T cells (both CD8+ and CD4 + T cells) are mainly involved in patrolling, conducting continuous surveillance and deploying quick recruitment to the site(s) of infection [80,81] thereby providing a rapid, effective, specific and durable protection against subsequent malaria infections. In *P. berghei* and *P. yoelii* studies CD8 + T memory cells have been described as CXCR^hi^CXCR6^hi^CD62L-CD69+ liver-resident cells (T_RM_) or CXCR3^lo^CXCR6^lo^CD44+CD62L-CD122- circulating effector (T_EM_) cells or CD44+CD62L + CD122+ central memory (T_CM_) cells [[82], [83], [84]]. The effector immune responses of these different memory CD8 + T cells subsets are species specific [82].

As expected mice studies have shown that a high proportion of circulating CD8 + T memory cells are T_EM_ with a smaller proportion of T_CM_ having also been observed [84,85]. In terms of their functional roles, T_EM_ have been observed to induce effector functions while T_CM_ tend to respond to sporozoites challenge in a delayed manner and by producing short-lived IFN-γ [72,84]. As such a higher population of T_EM_ cells is necessary for effective long-term protection [85,86]. In contrast, T_RM_ are non-circulating subset associated with protection to re-infection with sporozoites [87].

Recent *in vitro* studies seem to suggest that the patrolling and effector activities of *Plasmodium* specific T_RM_ is dependent upon Lymphocyte Function-Associated Antigen-1 (LFA1)- Intracellular Adhesion Molecule 1 (ICAM1) interaction [88]. Therefore, despite their reduced ability to recirculate, T_RM_ cells are crucial as part of the first line protective response against malaria infection but also in recruiting other immune cells to the site of infection [88].

The effector mechanisms used by CD8 + T cells to eliminate liver stage parasites are still not fully understood but some studies have shown that IFN-γ produced by CD8 + T cells has a potent inhibitory effect on the development of malaria parasites during the liver stage. CD8 + T cells are known to recognise cognate epitopes on infected hepatocyte MHC-1 and cluster around these cells [89]. Murine studies have shown increased CD8 + T cell effector mediators such as the cytokines IFN-γ and TNF-α, TRAIL, FAS Ligand and granzyme [31,80,90,91]. Surprisingly CD8 + T cells lacking perforin, FAS ligand have been observed to still be capable of eliminating *P. yoelli* and *P. bergheii* infected hepatocytes [70]. Further studies have shown that eliminating NK cells greatly reduces the protective effect of CD8 + T cells. This finding suggests that NK cells play an intermediary role for CD8+ cells [92].

### 4.3 CD4+ T cells in *P. falciparum* malaria

Work in murine models has shown that CD4 + T cells play an important role in protective immunity to erythrocytic stages of the infection. Mice without CD4 + T cells exhibited significantly higher parasitaemia upon being infected with *P. chabaudi* compared to controls and were unable to reduce parasitaemia during the course of infection [93]. In humans, CD4 + T cells play a major role in regulating immune response to *P. falciparum* infection after they were observed to inhibit parasite growth *in vitro* [94].

*In vitro* stimulation of CD4 + T cells with *P. falciparum* malaria antigens result in proliferation of CD4 + T cells from peripheral blood of individuals who have never been exposed to malaria infection before [95] and IFN-γ secretion, neither of which correlated positively with levels of serum antibody against corresponding antigens [96]. In other experiments, *P. falciparum* antigen stimulated IL-4 secretion CD4 + T cells correlated with neither lymphocyte proliferation nor IFN-γ release but with concentrations of relevant serum antibodies [97]. These two observations suggest that human immune response is controlled by distinct CD4 + T cell subsets that correspond to Th1 and Th2 cells and that both helper and effector functions of CD4 + T cells contribute to malaria immunity [98].

CD4 + T cells are required to help B cells produce antibodies for parasite clearance. They also produce cytokines that amplify the phagocytic and parasitocidal response of the innate immune system as well as regulating this response later on to limit immunopathology [99]. The role of CD4 + T cells in protective immunity against malaria parasite liver stages is achieved when they directly inhibit the development of the parasites and indirectly contribute towards the function of CD8 + T cells [100]. CD4 + T cell clones derived from peripheral blood mononuclear cells (PBMCs) also respond to a range of bacterial, viral and fungal preparations upon stimulation with *P. falciparum* parasite antigen. This observation supports the idea that memory T cells with a range of specificities may be maintained by cross-reactive stimulation [99].

A subset of CD4 + T cells known as T follicular Helper cells (Tfh) has been identified to play a crucial role in malaria infection [101]. Tfh cells are conventionally defined by the expression of Bcl6, CXCR5, PD-1, ICOS, SAP, CD40 L, TCF-1 and PSGL1 [102] and the CXCR5 is a well-known B-cell zone homing marker [103,104]. A circulating distinct subset of Tfh (cTfc) cells has been detected in peripheral human blood [105]. Malaria studies in mice models have shown that CD4 + T cell intrinsic Bcl6 signalling is required for induction of Tfh responses [106], and that the cytokines IL-6 and IL-21 are also required [107,108] whereas the presence of Type 1 IFN tended to compromise Tfh cells and Tfh-mediated GC responses [109]. More importantly, studies in humans have shown that during *P. falciparum* malaria infection activation of Th2-cTfh cells correlated with the development of functional antibodies required for protective immunity [110].

Tfh cells have been shown to be potent inducers of antibody production [111] which activate B cells within the germinal centres to generate high-affinity antibodies and memory B cells (MBCs) response [112,113]. The MBCs in turn produce long-lived plasma cells which maintain circulating antibodies in various diseases including malaria [114]. It is this link between Tfh cells and long-lived plasma cells which makes the former an ideal target for improving vaccine efficacy [115].

Studies in mice models, both *P. yoelli* and *P. bergeii*, have shown that malaria infection inhibits the establishment of germinal centres in the spleen and induce high frequency of Tfh cell precursors but results in impaired Tfh cell differentiation. However, blockade of TNF and IFN-g or Tbet deletion resulted in the restoration of the Tfh cell differentiation and GCs responses [116,117]. Reports of human studies have reported that *P. falciparum* malaria activates cTfh cells with increased expression of ICOS, HLA-DR, CD38 and Ki67 observed during acute disease compared to post-treatment with activation restricted to Th1-cTfh (CXCR3+) subsets [118]

Since Tfh cells seem to play such a crucial role in antibody development, some have proposed that this CD4 + T cell subset should be targeted as a potential route of improving vaccine efficacy [115]. In fact a recent study has shown that an early induction of functional IL-21secreting CSP-specific peripheral Tfh cell subset is one of the factors that improves the efficacy of RTS,S/AS01 when a delayed fractional dose (DFD) schedule was adopted [118]

### 4.4 B cells in *P. falciparum* malaria

B cells are the main lymphocyte subset that produces antibodies. Earlier work in mice models showed that B cell knockout mice were unable to eliminate parasites completely during primary acute infection with malaria parasite *P. chabaudi chabaudi* (AS strain) [119]. The mice ended up developing chronic relapsing parasitaemia instead. However, injection of B cells from immune donors into the chronically infected B cells knockout mice enabled them to clear their infection within a week, suggesting that B cells are required for final parasite clearance [120]. Other investigators have demonstrated B cell knockout mice retaining a predominantly CD4 + Th1-like response to malarial antigens throughout a primary infection. Surprisingly, the adoptive transfer of B cells resulted in a Th2 response in recipient mice, suggesting a role played by the B cells in the regulation of CD4 + T subset in response to malaria infection [121].

Further studies have shown that the long-lived plasma cells or short-lived plasma cells that result from differentiation of memory B cells (MBCs), which die off once the pathogens are eliminated, are the main source of the antibodies upon reinfection [122,123]. During an infection, antigen-specific B cells enter a germinal centre and differentiate into MBCs and long-lived plasma cells (LLPCs). LLPCs are terminally differentiated and maintain baseline levels of antigen-specific antibodies for years [123] whereas, antigen-specific MBCs are usually quiescent but rapidly differentiate into short-lived plasma cells (SLPCs) during reinfection that boost the level of antigen-specific antibodies until the pathogen is eliminated [122,124]. MBCs have been detected in some individuals sixteen years after malaria exposure but in the absence of circulating antibodies [125]. However, the observations that high titres of antigen specific antibodies measured during the malaria season decreased significantly during the dry season [126] seem to suggest that any high antibody levels are more likely the result of SLPCs after differentiation from MBCs.

### 4.5 Gamma/delta (γδ) T cells

Based on their response to infection, γδ T cells are generally considered as a bridge between the innate and adaptive immune response. Mice deficient of αβ T-cells immunised by the bites of irradiated *plasmodium*-infected mosquitoes were able to mount a response that confer partial protective immunity against sporozoites challenge [127]. When γδ T cells were depleted from these mice the protective immunity was almost completely abolished indicating that γδ T cells have some capacity to inhibit parasite development at liver stage and contribute to protective immunity in these mice [127]. The Vγ9Vδ2 subset of γδ T cells has been observed to increase in proportion and numbers in individuals presenting with malaria symptoms with the highest proportions observed in severe malaria [128]. The response of γδ T cells to stimulation *in vitro* with malaria antigens is characterised by proliferation as well as production of cytokines including IFN-γ, IL-1β, and TNF-α which have been associated with protection against malaria disease [129,130].

### 4.6 Natural killer (NK) cells

NK cells are a lymphocyte lineage with effector activities similar to cytotoxic T lymphocytes. They are a crucial first line of defence against pathogens due to their ability to exert their activity without prior sensitisation by antigen [131]. They increase in numbers during malaria infection and have the potential to lyse *P. falciparum*-infected erythrocytes *in vitro* [36,132].

Although these cells are usually associated with the innate immune system, their depletion proposes a role in antigen-specific acquired immunity to *P. falciparum* malaria. NK and T cell cells are major producer of IFN-γ driven by IL-12 in parasitic and bacterial models, essential for protective immunity [132]. NK cells are found in blood, secondary lymphoid organs and in peripheral non-lymphoid tissues. In non-immune donors, NK cells are among the first cells in peripheral blood to produce IFN-γ in response to *P. falciparum* infected erythrocytes [39,57]. Direct sensing of *P. falciparum* infection by NK cells induces their production of the pro-inflammatory chemokine IL-8 suggesting a role for the NK cells in the recruitment and activation of other cells during malaria infection [132].

### 4.7 Regulatory t cells

CD4+CD25+ T cells are a subset of T cells which control unregulated immune responses and have the capacity to limit activation, proliferation and effector function of both CD4+ and CD8 + T cells [133]. Regulatory T cells express high levels of transcription factor *Foxp3* in contrast with conventional CD4 + T cells. These cells are now known to play an immune suppression role in experimental malaria [134] and have been shown to expand in the spleen of *P. berghei* infected mice [135] and have also been found in higher proportions at different stages of human *P. falciparum* malaria [136,137]

## 5 The role of other immune factors in malaria

### 5.1 Cytokines

Cytokines, cell-derived polypeptides, involved in mediating inflammation are major determinants of the state of cellular activation and systemic responses to inflammation. Most of these factors are multifunctional and elicit their effects locally or systemically in an autocrine or paracrine manner [138]. Produced by such a diverse range of cells such as lymphocytes, monocytes, macrophages, fibroblasts, neutrophils, endothelial cells or mast cells [139], cytokines have been implicated as key determinants of malaria severity and outcome [140]. Some investigators have suggested that the balance between pro-inflammatory (TNF-α, IFN-γ, IL-6 and IL-8) and anti-inflammatory (IL-4, IL-10) cytokines determines the degree of malaria parasitaemia, level of anaemia, clinical severity, presentation and outcome [141].

### 5.2 Antibodies

Antibodies are crucial in protection against malaria with their role clearly demonstrated through experimental evidence of how transferred immunoglobulin from malaria immune adults to malaria naïve individuals offers passive protection [142,143]. Even maternal antibodies play a protective role in infants aged less than 6 months [144,145]. Antibodies are believed to provide the first line of defence by targeting a range of antigens expressed by various blood stages of the parasite. The antibodies are known to act against sporozoites invading the liver but also block merozoites from infecting erythrocytes and opsonise merozoites for uptake by phagocytes and antibody-dependent cellular inhibition [[146], [147], [148], [149], [150]].

Overall, studies on the protective associations for antibodies targeting merozoites have reported varying results [148,[151], [152], [153], [154]]. While some have provided evidence supporting the role of specific antibodies in protection, others have found little evidence supporting specific antibodies in protection or even an increased risk of symptomatic malaria. These differences may be explained by study design regarding the participant's age, malaria transmission intensity, and the level of immunity in the population [155,156]. Some investigators have recently compared anti-merozoite IgM and IgG dynamics following experimentally induced and natural malaria infection. They have reported that IgM was persistent in both young children and adults, similar to IgG, and is also likely to play a protective role by blocking merozoite invasion of erythrocytes in a complement-dependent manner [157,158].

Antibody levels are closely associated with recent malaria exposure in populations whose antibody levels have not yet reached predictive clinical immunity. As such, they are potential biomarkers of malaria risk as they may be used to identify individuals with the highest level of exposure to *Plasmodium* infection [159]. Antibodies specific for merozoite antigens, for example, have been extensively studied for this role of serological biomarkers of *P. falciparum* exposure or as biomarkers of immunity to help monitor changes in malaria transmission over time [[160], [161], [162]].

Antibodies responses to blood-stage malaria are required for inhibition of parasite invasion [[163], [164], [165]] suppressing the process of cytoadherence and sequestration to vascular endothelium by binding to parasite adhesion molecules (P*f*EMP-1) on the surface of iRBCs. They also neutralize parasite toxins like GPI thereby down-regulating inflammatory response, prevent fertilization of gametes and the production of zygotes, and induce the killing of gametes which are outside erythrocytes [148]. Longitudinal studies involving humans living in areas of high malaria transmission showed that repeated P. falciparum infections [166] can induce antibody responses to blood-stage antigens but that these responses are short-lived usually waning in a matter of days after acute infection.

The majority of studies examining the acquisition and role of malaria-specific antibodies in young children have been conducted in Africa. Data from some studies on antibody analysis points to exposure to a wide antigen diversity if one is to establish antibody-mediated protective immunity. Thus, a majority of strain-specific antibodies will only be able to recognize limited parasite strains [167]. As such, development of cross-reactive antibodies requires exposure to increased transmission intensity with multiple parasite variants overtime [[168], [169], [170]]. However, studies designed to assess the relevance of strain-specific and cross-reactive responses to clinical malaria immunity in endemic areas are complexed by unknown individual parasite exposure history.

The challenge in understanding the antibody repertoire is further complexed by the significant heterogeneity in *P. falciparum* transmission intensity [155,[171], [172], [173]], even within small geographical areas. Thus, exposure to *P. falciparum* is variable which also results in varied immunity acquisition. In residents of endemic areas, malaria infection induces strong humoral immune responses involving production of mainly IgM and IgG. Thus the main determinants of the type of IgG subclasses rely on the parasite antigenic properties rather than host factors [171,172].

Contrary to popular findings in older children and adults, high antibody levels in young children are not associated with protection from malaria; instead, they are typically associated with an increased risk of malaria. In order to ensure protection from malaria in younger children, some have proposed that there is a protective threshold of antibody levels that need to be reached [159]. Overall, antibodies against P. falciparum blood stages invariably appear during acute malaria episodes and rapidly increase in titres. Such significant changes in antibody levels propose a possible new approach to malaria screening, where, in combination with molecular diagnostics, a declining antibody titre may be considered as an indication of waning immunity against malaria. [157].

Malarial infections are also associated with elevated levels of total IgE and IgE anti-malarial antibodies. IgE elevation appears to be associated with malaria pathogenesis as the blood concentrations are significantly higher in CM patients than those with uncomplicated malaria [121,122]. Defining the role of specific antibodies in *Plasmodium* infections has several benefits, some of which include providing insight into how vaccines based on specific antigens may work. It may also enable the identification of potential endpoints for measuring the efficacy of vaccines in a clinical trial. Antibody detection can also be helpful for the retrospective differential diagnosis of malaria in non-immune travellers from endemic areas who report an episode of malaria-related fever [157,174,175]. Considering that antibodies have been shown to be essential in the success of vaccines’ response against other infectious diseases such as measles and TB, more effort should be extended to understanding how the humoral immunity against malaria can achieve robustness and longevity.

## 6 Progress on malaria vaccine development

A safe and protective antimalarial vaccine comprising of irradiated *P. falciparum* sporozoites was first successfully administered to humans back in 1973 [176]. Various attempts at novel vaccine candidates [177] have not been satisfactory. Due to the complex parasite life cycle, three distinct vaccine development approaches being explored are based on the three distinct stages in the parasite life cycle, focused on isolating and delivering antigens-specific vaccines instead of live attenuated vaccine [177].

### 6.1 Pre-erythrocytic vaccines candidates

These vaccine candidates (Fig. 1) are designed to elicit a robust immune response that would prevent the sporozoites from invading hepatocytes or destroy infected liver cells [177,178]. Considering that sporozoites can reach liver cells within 30 min after being injected by the mosquito, the challenge is for the immune system to act equally fast to impede their reaching their target. The RTS,S/ had proven to be the most successful candidate in this category as of 2020. The radiated circumsporozoite protein (CS) fused with a Hepatitis B surface antigen has proven to be immunogenic conferring some protection especially in young children aged five or younger and is now being tried in three African countries [10,179].

Although RTS,S has shown a median efficacy of 55.8 % in advanced clinical trials that had recruited African children [179], a new anti-malarial circumsporozoite protein-based vaccine, R21 with a Matrix-M™ (MM) adjuvant, has shown as high as 77 % efficacy at high-dose adjuvant groups in preliminary clinical trials conducted in Burkina Faso recently [180]. If subsequent trials result in these levels of efficacy or higher, the introduction of the R21/MM vaccine could prove to be the turning point in the fight against malaria.

Following the recent success story of mRNA-based vaccines against SARS-CoV-2, a number of investigators are now exploring if a similar approach could prove to be equally successful against malaria. A recent study [181] has shown some very promising results with an mRNA based vaccine which, similar to RTS,S relies on *P. falciparum* circumsporozoite protein (PfCSP) to generate an immune response. However, unlike RTS,S, instead of administering a version of the protein directly, this vaccine introduces the mRNA specific for the PfCSP which instructs the cells to synthesize their own circumsporozoite protein that triggers protective response against malaria [181]. Since this approach interrupts the malaria infection at a stage before the parasite reaches the RBCs, results in the mice models show the mRNA confer sterile protection against *P. bergheii* making it a very promising vaccine candidate for humans.

### 6.2 Erythrocytic vaccine candidates

These blood-stage vaccine candidates (Fig. 1) including PfRH5, are designed to block the rapid invasion and asexual reproduction of the parasite in RBCs. Since the blood stage is the time when malaria-related symptoms manifest with well over 40,000 merozoites released for each infected hepatocyte. An ideal blood-stage vaccine would aim to reduce the number of merozoites infecting RBCs rather than completely block their replication [179]. This being the case, currently there are no blood-stage vaccine candidates that have been as successful as the RTS,S vaccine.

### 6.3 Transmission blocking vaccine (TBV)

TBVs (Fig. 1) target the sexual reproduction stages in the mosquito gut to stop further spread of the parasite. This is an indirect approach to a vaccine since the individual who gets the parasite is not protected but rather prevents subsequent infections [178,179]. The *Pfs*25-EPA vaccine candidate is designed on the basis that those vaccinated will produce specific antibodies against this antigen so that if a mosquito feeds on this person it will take up some of these antibodies into its stomach. Once there, the antibodies will encounter the antigen, enabling them to interfere with development and kill the parasite [179].

## 7 Conclusions

Although the RTS,S/AS01 vaccine has thus far shown some promising results [10,180], the search for a safe, more efficient and effective malaria vaccine still continues. The fact that little is known about correlates for immunity against malaria makes the vaccine development process difficult and costly. Most experts seem to agree that the most feasible way forward is probably to combine multiple approaches although individual stage vaccines will still need to show significant and acceptable efficacy levels on their own before they are considered for combination with other vaccine candidates.

## Authors’ contributions

The whole manuscript was prepared and edited by all the authors

## Data availability

No data will be shared because these are indicated in the reference section

## Declaration of Competing Interest

The authors declares no competing interests
